# Unveiling the masquerade: renal cell carcinoma in a child mimicking an xanthogranulomatous pyelonephritis

**DOI:** 10.11604/pamj.2025.52.5.48210

**Published:** 2025-09-02

**Authors:** Nirlipta Swain, Shakti Sagar

**Affiliations:** 1 Department of Pathology, Jawaharlal Nehru Medical College, Datta Meghe Institute of Higher Education and Research, Sawangi (Meghe), Wardha, Maharashtra, India

**Keywords:** Xanthoma cells, fibrosis, bear paw, foamy histiocytes

## Image in medicine

Xanthogranulomatous pyelonephritis (XGPN) is a rare, aggressive form of chronic kidney inflammation, most commonly associated with long-standing urinary tract obstruction and renal calculi. It arises from obstructive uropathy that fosters recurrent infections and inflammation, leading to progressive fibrosis and destruction of renal parenchyma. Histologically, XGPN is distinguished by the replacement of renal tissue with lipid-laden macrophages (xanthoma cells) and dense chronic inflammatory infiltrates. Though predominantly seen in adults, XGPN is even less common in the pediatric population, with an incidence of about 1.4 cases per 100,000 people annually. This case describes a 12-year-old boy with a six-month history of fever, pain in the right flank, and recurrent urinary tract infections. Physical examination revealed right renal angle tenderness. Laboratory tests showed leukocytosis, anemia, thrombocytosis, elevated inflammatory markers, and pyuria. Computed tomography urography revealed a significantly enlarged right kidney (16.1 x 9.8 cm) with a staghorn calculus (2.6 x 2.3 x 1.6 cm), dilated calyces, and a “bear paw” appearance-classic signs of XGPN. The child underwent right radical nephroureterectomy. Gross examination showed loss of cortico-medullary distinction, pelvicalyceal system dilatation, thinned cortex, and parenchymal replacement by yellow, fatty nodules. A large staghorn calculus with hemorrhage was found in the renal pelvis. Microscopic analysis confirmed widespread fibrosis, hyalinized glomeruli, and dense inflammation with foamy histiocytes throughout the kidney. Following diagnosis, the patient was advised to undergo annual imaging of the remaining kidney, and the family was instructed to monitor for recurrent urinary symptoms or systemic signs of infection.

**Figure 1:**
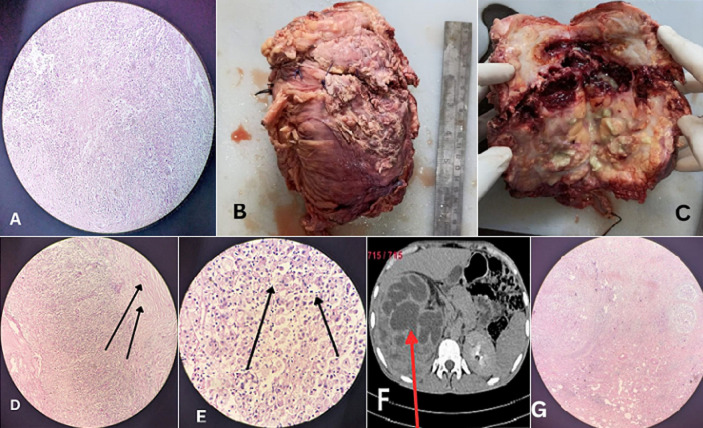
A) foamy histiocytes with dense chronic inflammatory infiltration 10x, H&E; B) gross right nephrectomy specimen showing an enlarged kidney with increased perinephric fat; C) cut section of the right kidney showing loss of cortico-medullary distinction, dilated pelvicalyceal system extending to the cortex, multiple yellow fatty nodules replacing renal parenchyma, and a thinned cortex; D) bands of fibrosis (black arrow) with dense chronic inflammatory infiltration 40x, H&E; E) foamy lipid-laden histiocytes (black arrow) 40x, H&E; F) computed tomography (CT) urography showing an enlarged right kidney with a staghorn calculus and dilated calyces, producing a bear-paw appearance (red arrow); G) microscopy showing hyalinized glomeruli with dense chronic inflammatory cell infiltration replacing the renal parenchyma 10x, H&E

